# Predictors of perceived male partner concurrency among women at risk for HIV and STI acquisition in Durban, South Africa

**DOI:** 10.1186/s12981-016-0098-7

**Published:** 2016-03-08

**Authors:** Zakir Gaffoor, Handan Wand, Renée A. Street, Nathlee Abbai, Gita Ramjee

**Affiliations:** HIV Prevention Research Unit, South African Medical Research Council, Durban, South Africa; The Kirby Institute, University of New South Wales, Sydney, Australia

**Keywords:** Concurrency, HIV, Prevention

## Abstract

**Background:**

Women in sub-Saharan Africa continue to be at greater risk for HIV acquisition than men. Concurrency, viz. multiple sexual partnerships that overlap over time, has been studied as a possible risk factor for HIV transmission. The aim of this study was to identify predictors of perceived male partner concurrency among sexually active, HIV negative women.

**Methods:**

Socio-demographic and behavioural data from women enrolled in a biomedical HIV prevention clinical trial were assessed in relation to perceived male partner concurrency using the Chi squared test. Univariate and multivariate logistic regression was performed to assess the independent predictors of perceived male partner concurrency. Kaplan–Meier survival estimates were obtained for HIV and STI incidence in relation to male partner concurrency. A Cox Proportional Hazards model was used to assess the association between perceived male partner concurrency and HIV and STI incidence.

**Results:**

The results revealed that 29 % of women reported their male partners to be in concurrent sexual relationships, 22 % reported partners that were not engaging in concurrency, whilst 49 % reported not knowing their partners concurrency status. Older women, having never married, experiencing economic abuse, and women reporting individual concurrency, were found to be significant predictors of perceived male partner concurrency in the studied population. Perceived male partner concurrency was not found to be a significantly associated with incident HIV and STI infections in this analysis.

**Conclusions:**

The study provides insight into predictors of perceived male partner concurrency among women at high risk for STI and HIV acquisition. These results may inform the design of behavioural and biomedical interventions, to address the role of multiple sexual partnerships in HIV prevention.

## Background

In 2012, an estimated 35.3 million people were living with HIV/AIDS, with 2.3 million new infections reported globally [1]. The latter figure represents a substantial 33 % decline, when compared to the HIV incidence reported in 2001. Sub-Saharan Africa continues to bear the brunt of the epidemic, accounting for 70 % of all new infections in 2012, with a majority of these occurring in women [1]. A broad range of factors contribute to women in this region being at a greater risk for HIV acquisition when compared to men. These include established gender inequalities, gender-based violence, and lack of access to proven preventative and treatment options, in addition to behavioural and biological risk factors [1].

There is evidence to suggest that certain behavioural interventions may reduce the risk of HIV acquisition among women [1, 2]. An important risk factor for HIV infection is multiple sexual partnerships, and programmes that aim to reduce the number of partners have proven effective in some settings [2]. Concurrency is defined as multiple sexual partnerships that overlap in time, where sexual intercourse with one partner takes place between two acts of sexual intercourse with another partner [3]. Mathematical modelling studies have demonstrated that small increases in the prevalence of concurrent partnerships can lead to substantial increases in the rate at which HIV is spread within a defined community [4]. There is less empirical consensus, however, as to whether concurrency is associated with HIV infection or not [5–7]. Whilst some studies hypothesize that concurrency is a primary driver of the HIV epidemic in sub-Saharan Africa [8], inadequate sampling, statistical methodology and inconsistent interpretations of the definition of concurrency yield inconclusive results [7].

In the context of concurrent partnerships, a person is at risk for HIV acquisition as a result of their partners other sexual contacts [9]. For the partner who engages in concurrent sexual partnerships, risk derives simply from sex acts with more than one partner, irrespective of whether the extra-couple partners are serial or concurrent [7]. For these reasons, standard epidemiological methods are insufficient in determining the empirical evidence signature for concurrency as a risk factor for HIV transmission [7]. Despite this, a number of studies have investigated individual-level concurrency in relation to HIV status, using standard logistic regression analyses [7]. Studies that recruit couples, and measure concurrency in relation to HIV transmission, are more likely to provide a rigorous estimate of the impact of concurrency in generalized epidemics [7, 10].

Multiple sexual partnerships, inclusive of concurrency, remain an important aspect of investigation into drivers of the HIV epidemic in our region. The aim of this study was to ascertain factors that predicted perceived male partner concurrency among a cohort of women at risk for HIV infection.

## Methods

### Study population

The recruitment methodology for the clinical trial has been published previously [11]. The study was a phase III multi-site, double-blinded, placebo-controlled trial testing the safety and efficacy of a microbicide, for the prevention of HIV infection in women [11]. Briefly, the main eligibility criteria included being sexually active; HIV negative at screening; willing to provide written consent and follow study procedures; not pregnant with intention to maintain a non-pregnant status; and anticipated residence in the study area for a minimum of 1 year.

Women who were HIV-positive at screening were referred to local health care facilities for care and support. Women who seroconverted during the trial remained in the study and were provided with ongoing counselling and referral to local health care facilities for further care upon completion of the studies. All protocols and informed consent forms were approved by the Biomedical Research Ethics Committee (BREC) at the University of KwaZulu-Natal, as well as the various study-specific Institutional Review Boards (IRBs).

### Data collection

This secondary analysis was done on a study population that consisted of 1485 women from Durban (KwaZulu-Natal), of whom data for 1456 was available for analysis. Socio-demographic and behavioural data were collected via interviewer-administered questionnaires. The outcome variable in the analysis was women who reported on knowledge of their steady sexual partner having other sexual partners besides themselves. The latter was based on a question in the behaviour questionnaire at the screening visit, which was administered prior to the women receiving risk-reduction counselling [11]. For the outcome variable, only data from the baseline visit was available for analysis. Steady partner was defined as the same partner reported by the participant throughout the study duration. The question asked was “*Does your steady sexual partner have sexual partners besides you*?” The three possible responses to this question were “1. *Yes*; 2. *No*; and 3. *I don’t know*”. For the purpose of our analysis, we considered the male partners of female participants to be concurrent, if they were reported as having additional sexual partners at the time of completing the questionnaire.

With regard to marital status, women were asked a single question viz. “*what is your marital status*”, for which responses could be “*single, never married*”; “*living as married*”, “*legally married*”, “*single, divorced*”, “*separated*”, “*widowed*” and “*refused to answer*”. Data on polygamous marriage was not collected. The proportion of women who reported being divorced, widowed or separated were negligible (3 %), and did not affect the outcomes of the analysis. Whilst “living as married” could be interpreted as non-married, cohabiting women; or traditional marriage, this was not defined in the protocol. We therefore grouped women into “married” and “never married” categories, where “married” included women who reported being legally married and/or living as married. Women also reported their marital status based on their own self-definition, in response to the question.

Data on intimate partner violence (IPV) were obtained from a single question, and categorised into economic, emotional and physical abuse. Each category was described in the questionnaire, by way of providing examples of different types of abuse, before the participants’ responses were recorded, as follows: *“sometimes in relationships women are abused by their partners. The abuse can physical, like hitting or slapping, emotional like yelling, name*-*calling or threatening the children, or economic like taking away or not giving money. We would like to know if any of these things are happening to the women we speak to”.* Responses to this question did not affect the eligibility of women for enrolment into the study.

### Laboratory procedures

HIV infection was diagnosed with two different rapid blood tests for the detection of HIV antibodies (Oraquick HIV 1/2, manufactured in Thailand for Orasure Technologies, Bethlehem, PA, USA; Determine, Abbott Laboratories, Wiesbaden, Germany; and UniGold Recombogen, Trinity Biotech, Wicklow, Ireland), which were done concurrently. Positive or discordant HIV rapid were confirmed by HIV PCR RNA (COBAS AmpliPrep, COBAS AMPLICOR HIV-1 MONITOR Test, version 1.5, Roche Diagnostics, Indianapolis, IN, USA). Pregnancy testing for the Human chorionic gonadotropin (hCG) hormone was conducted on urine samples using the QuickVue One-Step hCG Urine Test, Quidel Corporation, San Diego, CA, USA). *Neisseria gonorrhoeae* and *Chlamydia trachomatis* were detected in endocervical swab samples using the PCR Roche COBAS Amplicor (Roche Diagnostics, Indianapolis, IN, USA). *Trichomonas vaginalis* was detected in a vaginal swab using the In Pouch assay (BioMed Diagnostics, Santa Clara).

### Statistical analysis

Frequency distribution and percentages were used to describe the socio-demographic and behavioural characteristics of the study population. The Chi square test was used to test for a significant relationship between categorical variables. Univariate and multivariate logistic regression was used to assess predictors of perceived male partner concurrency, where the outcome was dichotomised as follows: 0 = "No" or "Don’t know" and 1 = "Yes". All variables presented in Table 1 were considered and stepwise regression was used to construct the final multivariable model. In logistic regression, age, marital status, women’s individual concurrent partners, and economic abuse were factors which we included in the final multivariate model. Kaplan–Meier survival analyses were carried out to estimate the crude HIV and STI seroconversion rates. The date of seroconversion was estimated using the midpoint between the last negative and the first positive test result within the follow-up period. The association between perceived male partner concurrency and HIV acquisition, while adjusting for age, women’s individual concurrent sexual partners, marital status, and economic abuse, was assessed using the Cox Proportional Hazards model. Similar analysis was conducted for STI incidence, where time to incident STI was defined as the time to first diagnosis with chlamydia, gonorrhoea, or trichomoniasis. In the final multivariate Cox model for STI incidence, we adjusted for women’s age, age of steady partner, forced sex, women who changed partners during the study, and diagnosis with any STI at screening. STATA Release 10.0 (Stata Statistical Software: Stata Corporation, College Station, Texas, USA) was used to conduct analysis of the data.

**Table 1:** Demographic and behavioural characteristics and their association with perceived male partner concurrency among a cohort of women in Durban, South Africa

	Total N = 1456 (100 %)	Steady sexual partner has another partner			p value
		Yes N = 425 (29 %)	No N = 322 (22 %)	I don’t know N = 709 (49 %)	
Age groups (years)					<0.001
<20	185 (100.0)	41 (22.2)	31 (16.8)	113 (61.1)	
20–24	392 (100.0)	104 (26.5)	65 (16.6)	223 (56.9)	
25–29	230 (100.0)	63 (27.4)	52 (22.6)	115 (50.0)	
30–34	188 (100.0)	57 (30.3)	43 (22.9)	88 (46.8)	
35–39	211 (100.0)	62 (29.4)	59 (28.0)	90 (42.7)	
40+	250 (100.0)	98 (39.2)	72 (28.8)	80 (32.0)	
Women has other partner besides steady partner					0.001
Yes	114 (100.0)	50 (43.9)	17 (14.9)	47 (41.2)	
No	1342 (100.0)	375 (27.9)	305 (22.7)	662 (49.3)	
Age of steady partner (in years older)					0.051
≤1 year older	375 (100.0)	111 (29.6)	88 (23.5)	176 (46.9)	
2–4 years older	484 (100.0)	136 (28.1)	94 (19.4)	254 (52.5)	
5–6 years older	247 (100.0)	61 (24.7)	59 (23.9)	127 (51.4)	
≥7 years older	342 (100.0)	117 (34.2)	81 (23.7)	144 (42.1)	
Marital status					<0.001
Married	382 (100.0)	90 (23.6)	141 (36.9)	151 (39.5)	
Never married	1074 (100.0)	335 (31.2)	181 (16.9)	558 (52.0)	
Pregnancy during the study					0.790
Yes	145 (100.0)	39 (26.9)	32 (22.1)	74 (51.0)	
No	1311 (100.0)	386 (29.4)	290 (22.1)	635 (48.4)	
Ever experienced forced sex					0.068
Yes	193 (100.0)	68 (35.2)	33 (17.1)	92 (47.7)	
No	1263 (100.0)	357 (28.3)	289 (22.9)	617 (48.9)	
Ever experienced economic abuse					0.033
No	1311 (100.0)	370 (28.2)	298 (22.7)	643 (49.0)	
Yes	145 (100.0)	55 (37.9)	24 (16.6)	66 (45.5)	
Ever experienced emotional abuse					0.101
No	1224 (100.0)	351 (28.7)	283 (23.1)	590 (48.2)	
Yes	232 (100.0)	74 (31.9)	39 (16.)	119 (51.3)	
Ever experienced physical abuse					0.302
No	1335 (100.0)	387 (29.0)	302 (22.6)	646 (48.4)	
Yes	121 (100.0)	38 (31.4)	20 (16.5)	63 (52.1)	
Sex for cash					0.306
No	1416 (100.0)	409 (28.9)	315 (22.2)	692 (48.9)	
Yes	40 (100.0)	16 (40.0)	7 (17.5)	17 (42.5)	
Steady partner circumcised					0.049
No	1111 (100.0)	308 (27.7)	244 (22.0)	559 (50.3)	
Yes	345 (100.0)	117 (33.9)	78 (22.6)	150 (43.5)	
Changed partner during the study					0.032
No	1307 (100.0)	371 (28.4)	300 (23.0)	636 (48.7)	
Yes	149 (100.0)	54 (36.2)	22 (14.8)	73 (49.0)	
Reported >3 coital acts in the 2 weeks prior to screening					0.730
No	917 (100.0)	268 (29.2)	197 (21.5)	452 (49.3)	
Yes	539 (100.0)	157 (29.1)	125 (23.2)	257 (47.7)	
Unprotected oral sex at last act					0.125
No	1238 (100.0)	374 (30.2)	270 (21.8)	594 (48.0)	
Yes	218 (100.0)	51 (23.4)	52 (23.9)	115 (52.8)	
Unprotected anal sex at last act					0.612
No	1376 (100.0)	405 (29.4)	305 (22.2)	666 (48.4)	
Yes	80 (100.0)	20 (25.0)	17 (21.3)	43 (53.8)	
Condom use at baseline					0.002
No	775 (100.0)	217 (28.0)	200 (25.8)	358 (46.2)	
Yes	679 (100.0)	208 (30.6)	122 (18.0)	349 (51.4)	
Any contraceptive use reported at screening					0.129
No	444 (100.0)	144 (32.4)	100 (22.5)	200 (45.0)	
Yes	1012 (100.0)	281 (27.8)	222 (21.9)	509 (50.3)	
Non-reversible contraception					0.007
No	1290 (100.0)	378 (29.3)	270 (20.9)	642 (49.8)	
Yes	166 (100.0)	47 (28.3)	52 (31.3)	67 (40.4)	
Hormonal contraception					0.016
No	898 (100.0)	280 (31.2)	207 (23.1)	411 (458)	
Yes	558 (100.0)	145 (26.0)	115 (20.6)	298 (53.4)	
Condom use as contraception					0.001
No	1169 (100.0)	330 (28.2)	283 (24.2)	556 (47.6)	
Yes	287 (100.0)	95 (33.1)	39 (13.6)	153 (53.3)	
Diagnosed with STI at screening					0.028
No	1151 (100.0)	319 (27.7)	267 (23.2)	565 (49.1)	
Yes	305 (100.0)	106 (34.8)	55 (18.0)	144 (47.2)	

## Results

Table 1 describes various socio-demographic, biological and behavioural factors that were evaluated for association with steady partner concurrency status. Twenty-nine percent of women reported their steady partners as having at least one other sexual partner besides themselves, whilst 49 % of women reported not knowing if their steady partner had other partners. There were no statistically significant associations between perceived steady partner concurrency status and pregnancy, forced sex, reporting emotional and physical abuse, sex for cash, reporting >3 coital acts in the 2 weeks prior to screening, unprotected oral and anal sex;, and any contraceptive use at screening. Significant associations were noted between perceived male partner concurrency and women’s age, partner age, women’s individual concurrency status, marital status, economic abuse, partner circumcision status, women changing partners during the study, condom use, contraception use and being diagnosed with an STI at screening.

With regard to age, a greater proportion of older women (35 %) reported their partner being engaged in concurrent relationships when compared to younger women (25 and 28 % in the <25 and 25–34 age groups, respectively); whilst more young women <25 years of age reported not knowing their partners concurrency status, when compared to older women (58 versus 37 % among women ≥35 years of age, respectively).

Forty-four percent of women who engaged in concurrent sexual partnerships themselves, also reported their steady partner as being concurrent. In comparison, 28 % of women who only reported one steady partner themselves, reported their partner as being in concurrent sexual relationships. A greater proportion of women who changed their steady partner at least once during the study, also reported their male partner as being in concurrent relationships (36 %), compared to the women who did not report their male partner as having additional partners (28 %). Thirty-seven percent of married women said their steady partner was not engaged in concurrent partnerships, compared to 17 % of never married women. Of note is that nearly 40 % of married women did not know if their steady partner was in a concurrent relationship, compared to 52 % of never married women. More women who reported economic abuse also reported male partner concurrency (38 %), compared to women who did not report economic abuse (28 %). A greater proportion of women with a steady partner that was circumcised, also reported them as being in concurrent partnerships (34 %), compared to women who reported a non-circumcised steady partner (28 %).

Thirty-one percent of women who reported their steady partner as being in a concurrent relationship, reported consistent condom use at baseline, whilst condom use at baseline decreased to 18 % among those whose partners were not reported to be engaged in concurrency. Fifty-three percent of women who had non-reversible contraception, also reported not knowing their partners concurrency status, compared to those who knew of their partner being concurrent or not (26 and 21 %, respectively). Similar trends were noted among women who reported hormonal and condom use as contraception.

Thirty-five percent of women who were diagnosed with any STI at screening reported male partner concurrency, compared to women who did not report having a male partner engaged in concurrency (18 %).

To further analyse and compare participant characteristics with perceived male partner concurrency, significant factors from Table 1 were analysed using univariate and multivariate logistic regression (Table 2). Older women (>35 years of age) were more than twice as likely to report having a steady partner that engaged in concurrent relationships, when compared to women <25 years of age [OR 2.19 (1.61–2.98), p < 0.001]. Women who reported a concurrent sexual partner themselves, in addition to their steady sexual partner, were almost twice as likely to report their steady sexual partner being in concurrent sexual relationships, when compared to women who reported a single steady partner [OR 1.77 (1.19–2.64); p = 0.01]. Women who reported never being married were similarly twice as likely to report male partner concurrency, compared to married women [OR 2.01 (1.48–2.72); p < 0.001], whilst women who reported economic abuse were one and a half times more likely to report male partner concurrency, compared to those who did not report economic abuse [OR 1.52 (1.06–2.20); p = 0.02].

**Table 2:** Predictors of perceived male partner concurrency, among women enrolled in the Carraguard™ trial in Durban, South Africa

Steady sexual partner has other partner				
	Univariate Analysis		Multivariate analysis	
	Odds ratio (95 % CI)	p value	Odds ratio (95 % CI)	p value
Age groups (years)				
<25	1		1	
25–34	1.19 (0.90, 1.59)	0.21	1.42 (1.06–1.91)	0.02
>35	1.58 (1.21, 2.07)	0.001	2.19 (1.61–2.98)	<0.001
Age of steady partner (in years older)				
≤1 year older	1			
2–4 years older	0.92 (0.69, 1.25)	0.63		
5–6 years older	0.78 (0.54, 1.12)	0.18		
≥7 years older	1.23 (0.90, 1.69)	0.18		
Concurrent sexual partner in addition to steady partner				
No	1		1	
Yes	2.01 (1.36, 2.97)	<0.001	1.77 (1.19–2.64)	0.01
Pregnancy during the study				
Not pregnant	1			
Pregnant	0.88 (0.059, 1.29)	0.52		
Ever experienced forced sex				
No	1			
Yes	1.35 (0.98, 1.86)	0.06		
Ever experienced economic abuse				
No	1		1	
Yes	1.55 (1.08, 2.21)	0.01	1.52 (1.06–2.20)	0.02
Ever experienced emotional abuse				
No	1			
Yes	1.16 (0.86, 1.57)	0.32		
Ever experienced physical abuse				
No	1			
Yes	1.12 (0.75, 1.67)	0.57		
Sex for cash				
No	1			
Yes	1.64 (0.86, 3.12)	0.13		
Steady partner circumcised				
No	1			
Yes	1.33 (1.03, 1.73)	0.02		
Marital status				
Married	1		1	
Never married	1.47 (1.12, 1.92)	0.01	2.01(1.48–2.72)	<0.001
Changed partner during the study				
No	1			
Yes	1.43 (1.00, 2.04)	0.04		
Reported > 3 coital acts in the 2 weeks prior to screening				
No	1			
Yes	0.99 (0.78–1.25)	0.96		
Unprotected oral sex				
No	1			
Yes	0.71 (0.50, 0.99)	0.04		
Unprotected anal sex				
No	1			
Yes	0.79 (0.47, 1.34)	0.39		
Condom use at baseline				
No	1			
Yes	1.13 (0.90–1.42)	0.27		
Any contraceptive use reported at screening				
No	1			
Yes	0.80 (0.62, 1.01)	0.07		
Non-reversible contraception				
No	1			
Yes	0.95 (0.66, 1.36)	0.79		
Hormonal contraception				
No	1			
Yes	0.77 (0.61, 0.98)	0.03		
Condom use as contraception				
No	1			
Yes	1.25 (0.95–1.65)	0.10		
Diagnosed with STI at screening				
No	1			
Yes	1.38 (1.06, 1.81)	0.01		

**Fig. 1:**
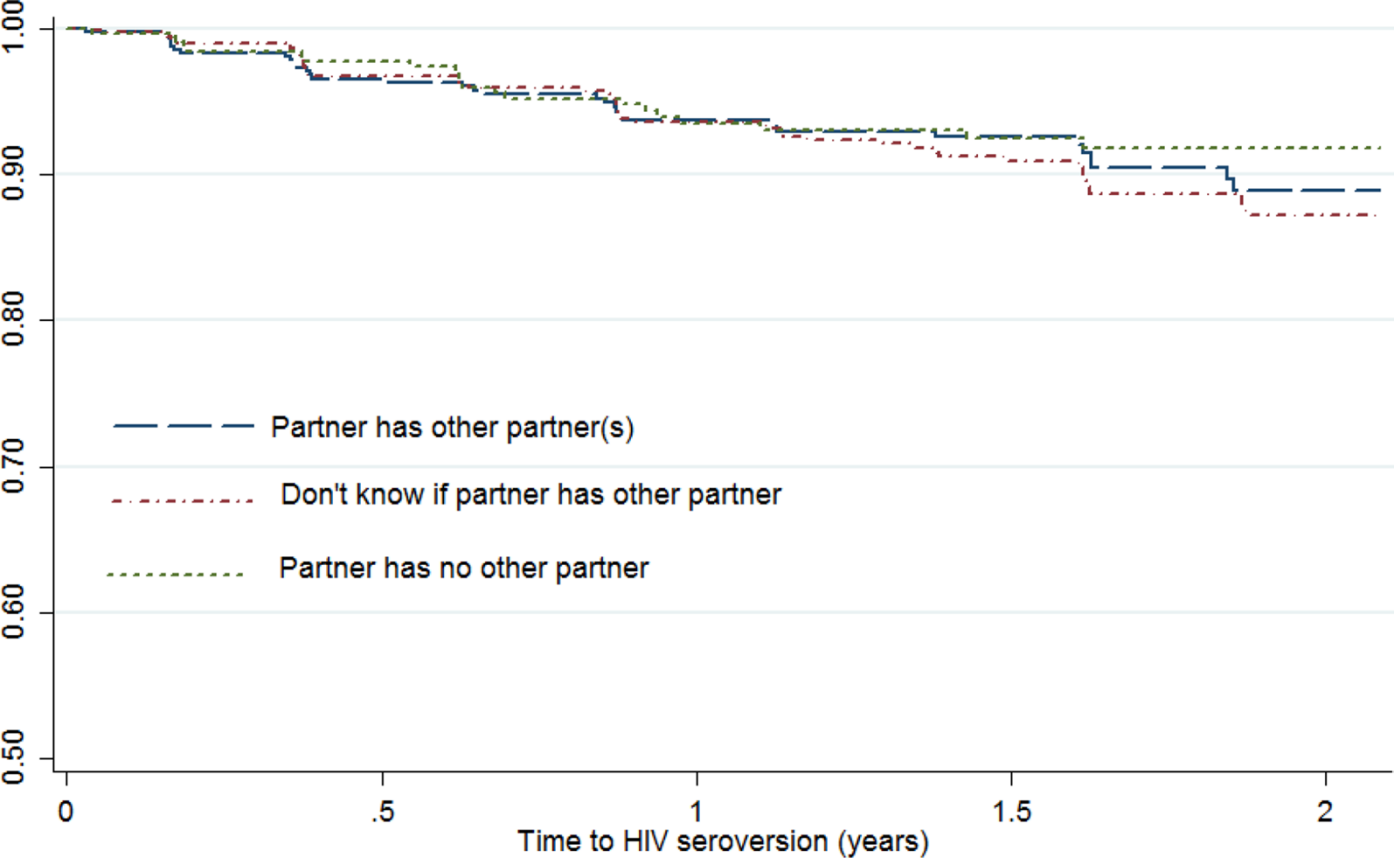
Kaplan-Meier survival curve for HIV incidence versus partner concurrency status, among a cohort of women in Durban, South Africa

**Fig. 2:**
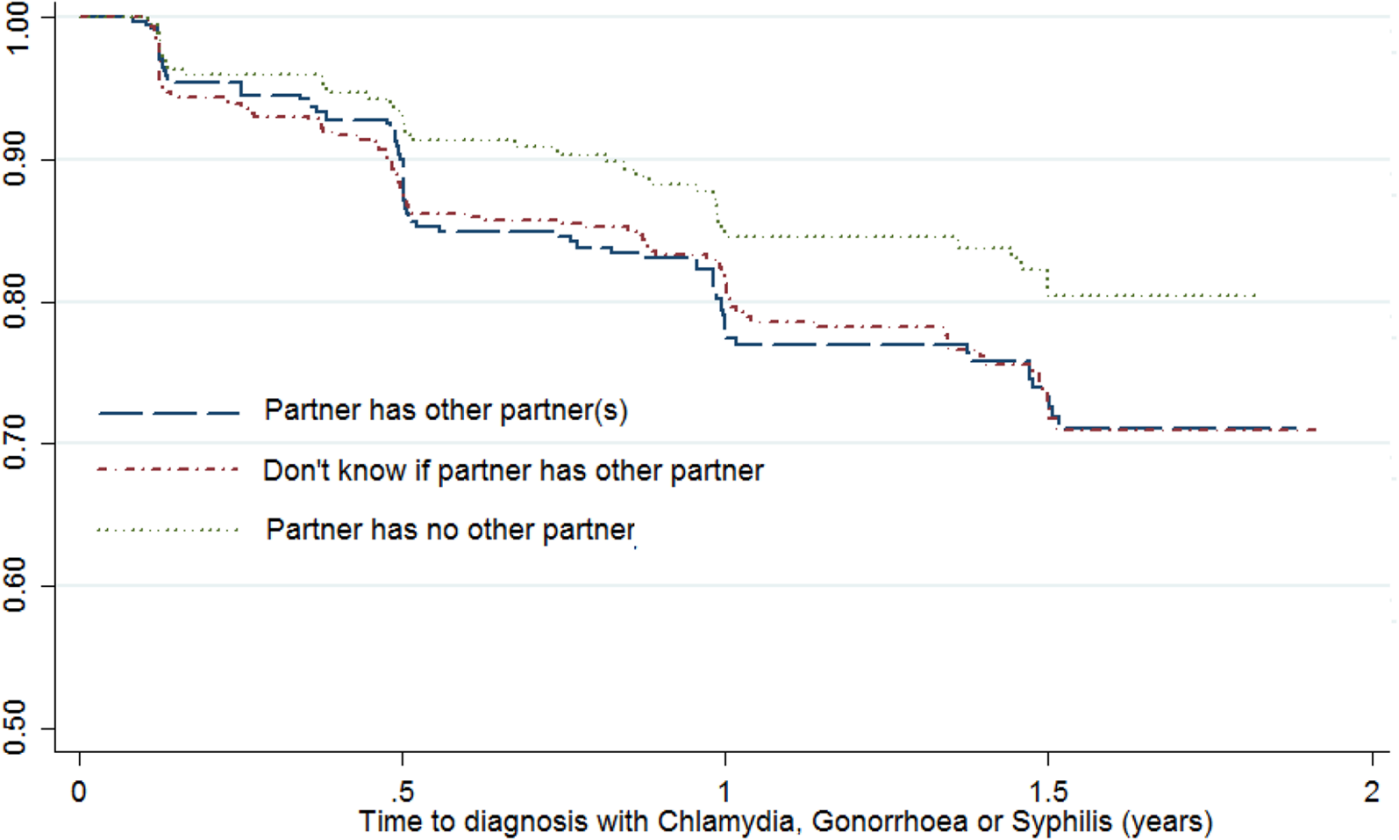
Kaplan-Meier survival curve for STI incidence versus partner concurrency status, among a cohort of women in Durban, South Africa

We examined the association between male partner concurrency and HIV and STI incidence (Figs. 1, 2; Table 3). According to the Kaplan–Meier survival estimates (Fig. 1), the largest number of HIV-1 seroconversions were observed among women that did not know if their partner had other partners. Approximately 10 % of women that were unaware of their partners concurrency status had seroconverted after being enrolled in the study for about 18 months. The overall crude HIV incidence rates associated with not knowing their partners concurrency status; knowing their partner to be in concurrent relationships; and not reporting a partner who engaged in concurrency was 6.5 per 100 person years (PY), 95 % CI 5.0–8.4; 5.9 per 100/PY (95 % CI 4.2–8.3;) and 5.0 per 100/PY (95 % CI 3.2–7.6), respectively (p = 0.583). In the univariate cox regression model for HIV incidence, the hazard ratios for women who reported a steady partner as engaging in concurrency, or reported not knowing, indicated a higher risk of HIV acquisition, however this result was not statistically significant [HR 1.17 (0.68–2.03), p = 0.5; and HR 1.30 (0.78–2.15), p = 0.30; respectively].

**Table 3:** HIV/STI incidence and its association with perceived male partner concurrency among women in the Carraguard™ trial in Durban, South Africa

	Total N (%)	Univariate analysis		Multivariate analysis	
		HR (95 % CI)	p value	HR (95 % CI)	p value
*HIV incidence*					
Steady partner has other partner					
No	322 (22)	1	–	1	–
Yes	425 (29)	1.17 (0.68–2.03)	0.55	–	–
I don’t know	709 (49)	1.30 (0.78–2.15)	0.30	–	–
*STI incidence*					
Steady partner has other partner					
No	322 (22)	1	–	1	–
Yes	425 (29)	1.53 (1.05–2.24)	0.02	1.35 (0.91–1.99)	0.125
I don’t know	709 (49)	1.51 (1.05–2.16)	0.02	1.20 (0.83–1.73)	0.320

The overall crude STI incidence rates (Fig. 2) for women who reported knowing their partner to be concurrent; those who did not know and those whose partners were not reported as engaged in concurrency, were 20.9 per 100/PY (95 % CI 16.8–26.0), 20.8 per 100/PY (95 % CI 17.3–25.0) and 13.5 per 100/PY (95 % CI 9.9–18.3) respectively (p = 0.04). The majority of women (>10 %) had acquired incident STIs after being enrolled in the study for approximately 6 months. In the univariate cox regression model for STI incidence, the hazard ratios indicated a significantly greater risk of STI infection among women who reported a steady partner in concurrent relationships, or reported not knowing their partners concurrency status, compared to those who reported a partner not engaged in concurrency [HR 1.53 (1.05–2.24), p = 0.02; and 1.51 (1.05–2.16) p = 0.02; respectively]. In the multivariate model however, this result was not statistically significant.

## Discussion

To our knowledge, this is one of the first studies from the Durban region to have investigated predictors of perceived male partner concurrency, among women at risk of HIV and STI acquisition. The effect of social desirability bias when responding to sensitive behavioural interviewer-administered questionnaires likely played a factor in this analysis, and must be taken into account when interpreting these findings. In response to the question about their partner having other sexual partners besides themselves, women may have felt social pressure when responding positively. Nearly 50 % of women did not know if their partner engaged in sexually concurrent partnerships. This finding may be relevant to understanding women’s risk perception for HIV acquisition, and may inform the design of HIV prevention strategies in this region. Further qualitative research is needed to understand the implications of this finding.

The prevalence of male partner concurrency reported in this study (29 %) is broadly similar to other studies conducted in Africa, but should be interpreted with caution, given that the data was obtained indirectly from the women [2, 5, 8, 10, 12–14]. Whilst we did not measure if their male partners sexual relationships overlapped in time as per the UNAIDS definition of concurrency, and given that the participants’ reported these men to be their steady partners throughout the study period, we consider our analysis a close approximation of true concurrency. It may therefore be feasible to include questions about sexual partners in routine surveys in order to identify groups that may be at risk for HIV acquisition [15].

Various studies have demonstrated that women and men in sub-Saharan Africa tend to under-report and over-report multiple sexual partnerships, respectively [16–19]. A nationally representative survey conducted by Steffenson and colleagues [19] among South African youth, showed that 34 % of women and 18 % of men reported their partner to be engaged in concurrent relationships. In the same study cohort, 25 % of men and <5 % of women reported concurrent relationships in the past year [19]. Our study measured women’s perceptions of their steady male partners concurrency status, and men were not questioned regarding their individual concurrency status. The concurrency prevalence estimates from this analysis must therefore be viewed in the context of a discordance, between perception of partner concurrency and actual concurrency measurements.

Our study shows that younger women (<25 years of age) are 50 % less likely to report their partner being concurrent, compared to women >35 years of age. After an extensive review of the literature, we were unable to find comparable studies, with a similar study design, where age of women predicted perceived male partner concurrency. Whilst previous studies have provided evidence for age-disparate relationships as being a possible risk factor for concurrency, and also for HIV acquisition among women in our local setting, we did not note a significant association in this report [20, 21]. It is important to note that women’s perceptions of their male partners concurrency status was measured in this analysis, preventing reliable comparisons with studies that directly measured male partner concurrency.

Whilst unmarried women were twice as likely to report a steady partner in concurrent relationships when compared to married women, it is notable that nearly 40 % of married women in this study knew of their partner having other partners. We did not differentiate the type of extra-couple partnership in this study (polygamous or not). Further research into the latter is required, given that some studies have reported culturally practised polygamy as being benign at the population level, in relation to risk of HIV transmission [10].

Intimate partner violence was observed in our study. In particular, women who said they experienced economic abuse were also twice as likely to report a male partner being in a concurrent relationship. Whilst different measures of IPV across studies may preclude direct comparisons, these findings are consistent with those reported elsewhere, where known risk factors for intimate partner violence include a male partner who engages in concurrent relationships [22].

Our study found that women who engaged in concurrent sexual partnerships themselves, were nearly twice as likely to, report their male partner as also engaged in concurrent partnerships. A study that measured concurrency among urban youth in Kenya, observed an association between perceived partner concurrency and individual concurrency, suggesting that some populations respond to partner concurrency (or perceptions there of) by engaging in additional concurrent partnerships [14]. Other investigators have reported changes in individual-level concurrency when corresponding perceived partner concurrency changes as well [20].

Consistent condom use was not significantly associated with perceived partner concurrency. This finding is consistent with that reported by Delva and colleagues [23], where no significant changes were noted in condom use and coital frequency during concurrent episodes that were measured among a study cohort in Cape Town, South Africa. However, the latter study was designed to measure changes in behaviour in response to relationship concurrency status over time, and may not be strictly comparable to our findings. Another South African report indicated less consistent condom use among those reporting concurrency [19]. Various other studies have demonstrated a variation in condom use with type of partner, either main partner or casual [24, 25]. We were unable to measure the nature of concurrent partnerships that men were perceived to be in, by women in our study. Nevertheless, other reports in similar settings have observed low levels of consistent condom use with a main partner, and more consistent condom use with additional casual partners, irrespective of overlap time.

While slightly higher HIV incidence rates were observed among women who knew of their partners being concurrent, or did not know, compared to those who did not report a steady partner as being in concurrent relationships, the association was not statistically significant. This is in keeping with studies that identified the index case for measuring concurrency as the partner of the individual, as opposed to other studies that found associations through logistic regression analysis, by measuring individual concurrency and HIV incidence [5, 7]. It is noted that 49 % of women in this study reported not knowing if their steady partner was engaged in concurrent sexual partnerships, however cox regression analysis showed no significant association between perceived male partner concurrency and HIV and STI incidence, irrespective of whether or not women responded definitively to the outcome variable. Nevertheless, given the similar HIV incidence rates among women who reported their steady partner to be engaged in concurrency, and those who reported not knowing, it is possible that women in both groups were at similar risk for HIV infection. A previous study by Drumright and colleagues (2004) also demonstrated an association with STI infection and not knowing a partner’s concurrency status. Our study was conducted among a population at high risk for HIV infection, and known to engage in high-risk sexual behaviours [26, 27]. In the absence of other possible confounders, it is likely that social desirability bias contributed to this study showing no statistically significant association between both HIV and STI incidence, and perceived male partner concurrency. Furthermore, reporting on the association between perceived partner concurrency and HIV incidence among women in this study is limited, given that the HIV status of the male partner was not confirmed by diagnosis.

A review of qualitative data regarding concurrency in sub-Saharan Africa suggests that the practise may be a deeply rooted cultural phenomenon that is accepted despite the high prevalence of HIV infection [2]. The role that concurrency may play in the transmission of generalised HIV epidemics such as that seen in our local setting cannot therefore be discounted, since concurrency is a subset of multiple sexual partnerships, the latter of which is a key driver of the epidemic. The results of our analysis does not support concurrency as being an important driver of HIV incidence in our high-prevalence setting. With regard to the latter, further research is needed that measures HIV status in dyad couples, in order to accurately assess male partner concurrency as a predictor of HIV acquisition among women.

In this study, we did not find a significant association between women who reported partners that were engaging in concurrent sexual relationships, and baseline STI prevalence. This finding differs from a recently published study by Weir and colleagues [15]. In addition, our study did not find a significant association between STI incidence and perceived male partner concurrency. Whilst some studies have found an association between specific STI prevalence and concurrency, there is limited information with regard to STI incidence in the context of perceived male partner concurrency [28, 29]. Investigating the association between perceived male partner concurrency and STI incidence is further complicated by the nature of the infectious period for the STI in question.

## Limitations

Our report had several limitations. Firstly, data were collected from women who presented themselves for screening in an HIV prevention trial. These women were actively recruited based on, among other inclusion criteria, their risk for HIV acquisition. The results may therefore not be generalizable to the local population as a whole. We were not able to measure concurrency per the guidelines recommended by UNAIDS, therefore the prevalence estimate of concurrency may not be strictly comparable to other reports in the field. Furthermore, varying definitions of what constitutes concurrency may also pose a challenge. The behavioural questionnaire used was interviewer-administered, and responses may have been subjected to social desirability bias. Furthermore, only data from the baseline visit with regard to measuring perceived male partner concurrency, were available for analysis. Thus, we were unable to ascertain any change over time, with regard to the outcome variable among women who reported on steady partner concurrency. Given the design of the clinical trial from which this secondary analysis was conducted, the HIV status of male partners were not accurately determined via standard diagnostic techniques at any time point during the study. As a result, it was not possible to determine whether HIV incidence was related to male partner concurrency, or to other risk factors not accounted for. Furthermore, we were unable to measure directly from male partners’, their experiences with concurrency.

## Conclusion

A high prevalence of perceived male partner concurrency (29 %) was reported by women in this study. Furthermore, we observed a high percentage of women who didn’t know if their partner engaged in concurrent sexual partnerships (49 %). Older women, never being married, experiencing economic abuse and women reporting individual concurrent sexual partnerships, were found to be significant predictors of perceived male partner concurrency in the studied population. The association between incident HIV and STI infections and perceived male partner concurrency was not found to be statistically significant in this study. A strength of this report is the relatively large sample size, as well as the fact that the study was conducted among a population with some of the highest HIV prevalence rates in the world. Further research is needed that recruits dyad couples, and measures concurrency using data collection methods that may be less subject to social desirability bias. The findings presented here also lend support for further research into behavioural and biomedical interventions that can address the role of multiple partnerships in HIV prevention.

## Abbreviations

UNAIDS: Joint United Nations Programme on HIV and AIDS
HIV: Human Immuno-Deficiency Virus
AIDS: Acquired Immuno-Deficiency Syndrome
STI: Sexually Transmitted Infection
RNA: Ribonucleic Acid
PCR: Polymerase Chain Reaction
hCG: Human Chorionic Gonadotropin
BREC: Biomedical Research Ethics Committee
IRB: Institutional Review Board
PY: Person Years
CI: Confidence Interval
